# On the Feasibility of Fan-Out Wafer-Level Packaging of Capacitive Micromachined Ultrasound Transducers (CMUT) by Using Inkjet-Printed Redistribution Layers

**DOI:** 10.3390/mi11060564

**Published:** 2020-05-31

**Authors:** Ali Roshanghias, Marc Dreissigacker, Christina Scherf, Christian Bretthauer, Lukas Rauter, Johanna Zikulnig, Tanja Braun, Karl-F. Becker, Sven Rzepka, Martin Schneider-Ramelow

**Affiliations:** 1 Silicon Austria Labs GmbH, Europastrasse 12, 9524 Villach, Austria; lukas.rauter@silicon-austria.com (L.R.); johanna.zikulnig@silicon-austria.com (J.Z.); 2 Microperipheric Center, Technical University Berlin, 13355 Berlin, Germany; marc.b.dreissigacker@tu-berlin.de (M.D.); Tanja.Braun@izm.fraunhofer.de (T.B.); Karl-Friedrich.Becker@izm.fraunhofer.de (K.-F.B.); martin.schneider-ramelow@izm.fraunhofer.de (M.S.-R.); 3 Materials and Reliability of Microsystems, Chemnitz University of Technology, 09111 Chemnitz, Germany; christina.scherf@enas.fraunhofer.de (C.S.); Sven.Rzepka@enas.fraunhofer.de (S.R.); 4 Micro Material Center, Fraunhofer Institute for Electronic Nano Systems, 09126 Chemnitz, Germany; 5 Infineon Technologies AG, 85579 Neubiberg, Germany; christian.bretthauer@infineon.com; 6 Fraunhofer-Institut für Zuverlässigkeit und Mikrointegration (IZM), 13355 Berlin, Germany

**Keywords:** microelectromechanical systems (MEMS) packaging, inkjet printing, redistribution layers, capacitive micromachined ultrasound transducers (CMUT), fan-out wafer-level packaging (FOWLP)

## Abstract

Fan-out wafer-level packaging (FOWLP) is an interesting platform for Microelectromechanical systems (MEMS) sensor packaging. Employing FOWLP for MEMS sensor packaging has some unique challenges, while some originate merely from the fabrication of redistribution layers (RDL). For instance, it is crucial to protect the delicate structures and fragile membranes during RDL formation. Thus, additive manufacturing (AM) for RDL formation seems to be an auspicious approach, as those challenges are conquered by principle. In this study, by exploiting the benefits of AM, RDLs for fan-out packaging of capacitive micromachined ultrasound transducers (CMUT) were realized via drop-on-demand inkjet printing technology. The long-term reliability of the printed tracks was assessed via temperature cycling tests. The effects of multilayering and implementation of an insulating ramp on the reliability of the conductive tracks were identified. Packaging-induced stresses on CMUT dies were further investigated via laser-Doppler vibrometry (LDV) measurements and the corresponding resonance frequency shift. Conclusively, the bottlenecks of the inkjet-printed RDLs for FOWLP were discussed in detail.

## 1. Introduction

Fan-out wafer-level packaging (FOWLP) has spurred increasing interest due to significant cost advantages over competitive technologies, increased interconnect density, as well as enhanced electrical and thermal package performance. With its roots in integrated circuit (IC) manufacturing technology, FOWLP has also recently gained a lot of attention for microelectromechanical systems (MEMS) and sensors packaging. Some examples for FOWLP of sensors including MEMS-based acceleration and pressure sensors, capacitive micromachined ultrasonic transducers (CMUTs), gas sensors and biomedical sensors were recently realized and reported [1,2,3,4,5,6,7].

Employing FOWLP for MEMS sensor packaging creates some unique challenges [2]. For instance, the thin and sensitive parts of MEMS components are incompatible with FOWLP processes such as laminating, molding, back-grinding and dicing. This leads to deterioration of component performance, i.e., a resonance frequency shift of a MEMS microphone, or membrane rupture, which are issues with mold encapsulation per se [7].

Moreover, some issues are emerging from the typical fabrication processes of redistribution layers (RDL) for fan-out packages, a combination of sputtering, photolithographic processes, etching, as well as electroplating. RDLs are typically metal interconnects used to provide power supply and route signals within the package and towards its periphery [8]. To ensure proper functionality of MEMS, it is essential to temporarily protect delicate sensing areas during RDL processing and ensure that temporary protection is entirely removed afterward. This complex procedure for the protection of thin film is also known as keep-out-zones (KOZ) processing on RDLs [2].

**Figure 1:**
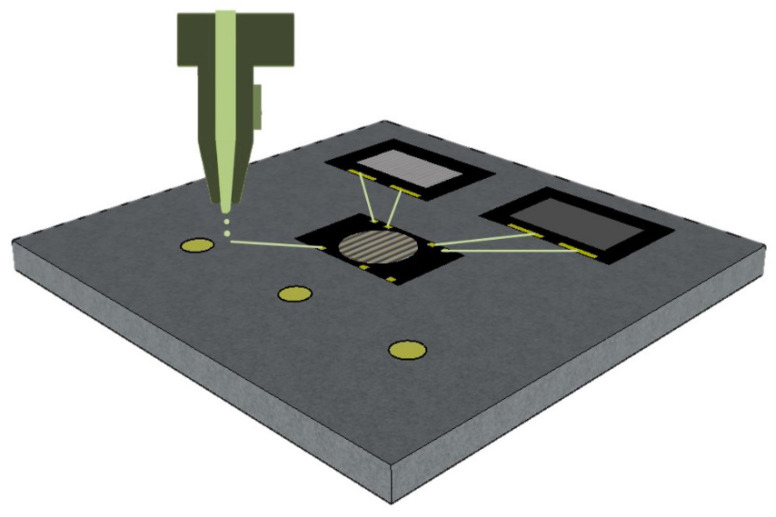
Schematic illustration of the fabrication process of redistribution layers in FOWLP by using drop-on-demand inkjet-printing.

In this study, an alternative FOWLP concept by implementing additively manufactured RDLs for CMUT array packaging was proposed. The printed RDLs served as an interconnect between capacitive microphones and speakers, operating in the ultrasonic domain, with corresponding application-specific integrated circuits (ASICs), which allow features such as touchless activation or control using gestures [9,10]. As schematically illustrated in Figure 1, metallic and dielectric structures can be deposited selectively and in a controlled volume via a drop-on-demand printing technology (e.g., inkjet, aerosol, electrostatic, electrohydrodynamic, etc.). The concept of inkjet-printed RDLs for FOWLP was introduced in our previous work [11]. Inkjet-printed circuitry was also evaluated for the fabrication of low-cost silicon [12] and organic interposers [13], which showed great potential for rapid prototyping and signal probing. Aerosol printed RDLs for 3D smart devices were also recently reported by Serpelloni et al. [14] as well as screen printed RDLs by Chia-Yen et al. [15].

Inkjet printing technology avoids long lithography procedures including global resist coating and sputtering, thus lower thermo-mechanical stresses are expected to be applied to the sensitive MEMS structures which will be assessed here. Additionally, long-term reliability of these printed RDLs for FOWLP will also be investigated and discussed.

## 2. Materials and Methods

In this study, an advanced R&D inkjet printer (PiXDRO LP50, Meyer Burger Technology AG, Gwatt, Switzerland) equipped with an industrial inkjet print-head (Spectra SE-128, Fujifilm Dimatix Inc., Santa Clara, CA, USA) was used. A commercial nanoparticle silver (Ag)-ink (Sicrys 115-TM 119, PV Nano Cell Ltd., HaZafon, Israel) with 50 wt.% metal loading and an average particle size of 120 nm (d90) was deposited at the operational jetting voltage of 120 V, printing frequency of 1000 Hz and at carefully adjusted jetting pulse duration profile. Consequently, droplets with an average volume of 20 picoliters and a velocity of 2 m/s were generated. The printing of Ag was performed at room temperature, while the substrate was heated up to 50 °C to facilitate the evaporation of solvents. After printing, the Ag lines were thermally sintered at 150 °C for 30 min. The electrical resistivity of the tracks after thermal sintering at 150 °C was 5.61 E-8 Ωm corresponding to ~33% of the conductivity of bulk Ag. For multilayering, several UV-curable dielectric inks were examined [16,17].

The morphology of the printed RDLs was characterized using scanning electron microscopy (SEM, Helios, Thermo Fisher Scientific Inc., Waltham, MA, USA) and a mechanical stylus profilometer (Dektak XT-A, Brucker, Elk Grove Village, IL, USA). In addition, the surface roughness of the substrates was measured using a white light interferometer (MSA 500, Polytec GmbH, Waldbronn, Germany) and calculated as defined in ISO 25,178 [18].

Capacitive acoustic sensor chips (Infineon Technologies AG, Neubiberg, Germany) with a size of 1.6 mm × 1.6 mm were utilized in this study [19,20]. The chip possesses a circular polysilicon membrane with a diameter of 0.9 mm and gold pads with a diameter of 0.1 mm. Silicon dummy chips with a size of 5 mm × 5 mm were also employed for reliability analysis. Chip placement was done using a Datacon 2200evo, while encapsulation by means of compression molding was done using a TOWA Y-120 to form an 8” wafer. The chips were assembled facedown onto a temporary carrier with laminated thermal release tape. Subsequently, compression molding, post-curing and release from the temporary carrier were performed, resulting in mold-embedded components into an 8” mold-wafer ready for inkjet printing. The liquid epoxy molding compound (EMC) had a filler load of 89 wt.% with a top filler cut of 75 µm, resulting in an overall coefficient of thermal expansion (CTE) of 7 ppm/K (*T* < *T*_g_).

The reliability performance of the printed tracks was evaluated by resistance measurements (Süss Microtec PM5, SUSS MicroTec, Garching, Germany). A temperature cycling test (−40 to 125 °C, 130 min per cycle; CTS CSR 60/600-5) was conducted for reliability analysis. Measurements were done at defined intervals during accelerated aging. Here two sets of experiments were planned to examine the difference between the single-layer and double-layer Ag printed lines as well as to explore the influence of the insulating layer beneath the Ag printed lines.

To investigate the introduced stresses due to the packaging process, laser-Doppler vibrometry (LDV) measurements were performed with both bare-die and mold-embedded CMUTs [21]. For the LDV measurements, a micromotion analyzer (MSA 400, MSA Safety, Cranberry Township, PA, USA) was used to conclude the first resonance frequency. The system provides a real-time velocity and displacement signal of vibration frequencies up to 1.5 MHz at maximal velocity amplitudes of 10 m/s. The excitation of the CMUT resonators was triggered by electrostatic forces. An electrostatic probe was connected to a high voltage excitation signal of up to 400 Volt and placed nearby the CMUT resonator at a distance of approximately 100 µm. The fringing field electrodes provided a strong electrostatic force; therefore, it was not necessary to electrically connect the samples to any potential.

## 3. Results and Discussion

### 3.1. Microfabrication

**Figure 2:**
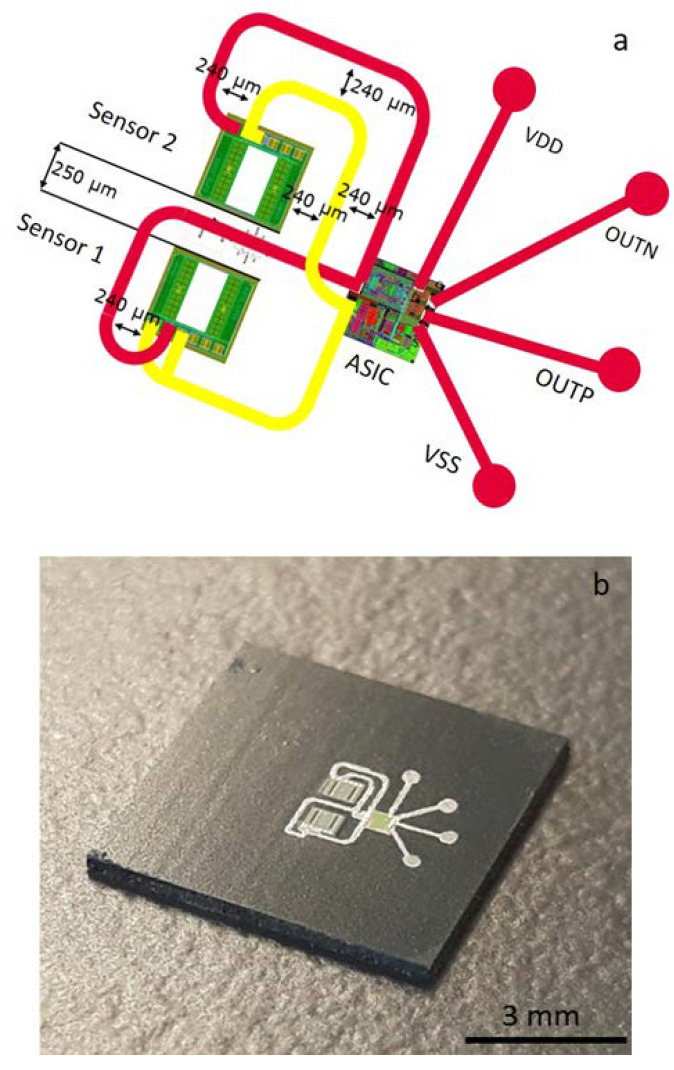
An example for the layout (**a**) and the final demonstrated FOWLP of two capacitive micromachined ultrasonic transducers (CMUTs) and an application-specific integrated circuit (ASIC) chip with inkjet-printed redistribution layers (RDLs) (Comprised of 2 layers of Ag printed lines (red and yellow) and an intermediate insulating printed layer (not shown)) (**b**).

**Figure 3:**
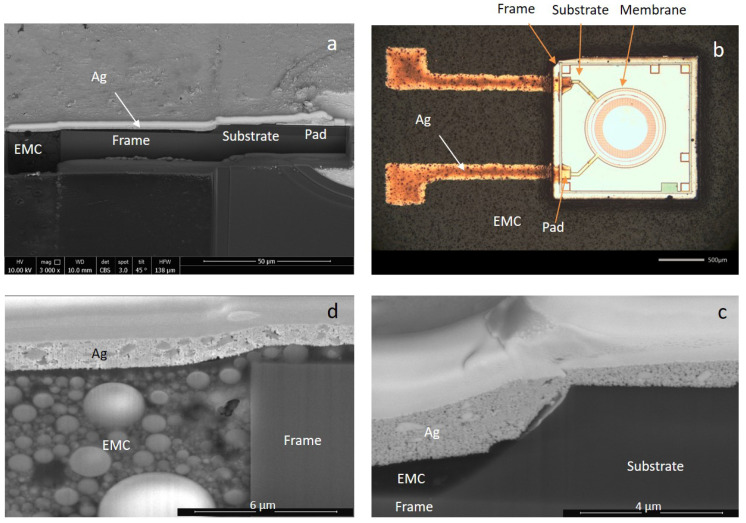
The cross-sectional (**a**) and the planar (**b**) view of the test vehicle comprised of a CMUT microphone with a sensitive membrane and two interconnecting Ag tracks. The critical interfaces between the CMUT substrate-frame (**c**) and CMUT frame-epoxy molding compound (EMC) (**d**) are shown in higher magnifications.

In Figure 2 and Figure 3, two realizations of inkjet-printed RDLs for CMUT FOWLP are depicted. As shown in Figure 2, a system-in-package (SiP) layout for FOWLP of CMUT consisting of two sensors and one ASIC was realized by inkjet printing. Here two Ag layers were ink-jetted over the metallic pads of components and EMC in a drop-on-demand manner. The dielectric layer was inkjet-printed between the two Ag tracks, which enabled multilayering. Depending on the number of input/output (I/Os), pad-diameter, and -pitch of the components, various line-widths for the Ag tracks (between 50 µm to 500 µm) were tailored. In fact, inkjet printing enabled us to customize RDL geometries and realize complex circuitries within a short time. 

**Figure 4:**
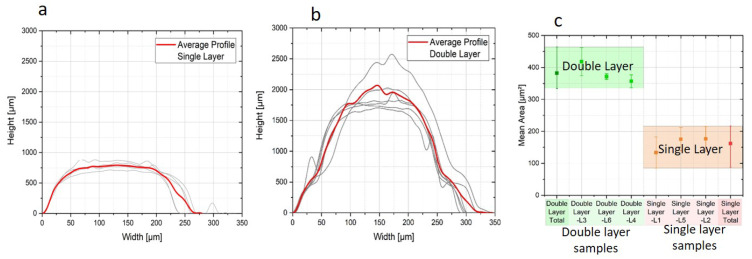
Surface profiles of the single-layer (**a**) and double-layer Ag tracks (**b**). The calculated areas under the profiles are compared in (**c**).

Since inkjet printing of particle-loaded inks is limited to inks with low viscosity (5–20 mPa·s [22]) and the major fraction of the ink is evaporated during curing, single layer printing usually yields a thin layer. It is noteworthy to state that the metal loading of the ink in this study was 50 wt.%. The average height of a single-layer Ag track was <1 µm. As a comparison, double-layer Ag lines were also prepared. Concerning the sequential processing of double-layer samples, the first layer was only dried before applying the second layer without an intermediate sintering step [23]. Here the average thickness reached ~2 µm, however, the line-width was also increased by up to ~20%. As an example, in Figure 4 the surface morphologies of the single-layer and double-layer Ag lines are compared. Here, Ag tracks with the line-width of 275 µm were aimed. As implied by this figure, the line-width of the double-layer extended to ~325 µm. The calculated area (under the surface profile) of the single-layer and double-layer Ag tracks is compared in Figure 4c. The results for 3 samples per kind are plotted here and averaged. It can be inferred that double-layer Ag tracks have on average double the area of the Ag materials when compared to single-layer tracks. 

**Figure 5:**
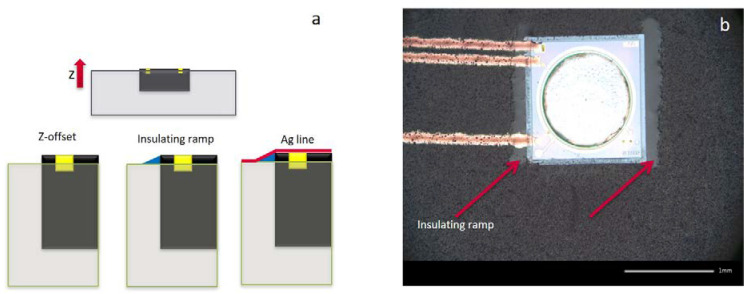
The schematic demonstration (**a**) of the inkjet-printed insulating ramp approach to smoothen the Z-offset and the corresponding optical image (**b**).

Figure 3 shows a rather simplified example of two inkjet-printed Ag tracks, which was selected as the test-vehicle for the performance analysis and fast signal probing. Here, the CMUT microphone possesses a sensitive membrane and the packaging-induced stress could be extracted by the resonance frequency shift. By observing cross-sectional images of the samples, it was observed that the chip surface is ~3–6 µm higher than the surface of the EMC. This sharp step was predicted to be the weak-spot for the long-term reliability of the RDLs. Figure 3c,d revealed two critical steps at the interfaces between CMUT frame/ CMUT substrate and EMC/ CMUT frame, respectively. In an attempt to smoothen the steps, dielectric inks were selectively inkjet printed on the edges of the components, forming a ramp. In Figure 5, one example of the printed dielectric ramp is depicted. By employing this approach, the Z-offset was smoothened and the sharp step between the chip and mold surface was diminished. 

### 3.2. LDV Analysis

As aforementioned, the mechanical stresses upon FOWLP of CMUTs can lead to either physical damage to the sensitive membrane or shifts of the acoustical properties of the CMUT due to the change of membrane stiffness. Accordingly, LDV measurements were performed on both bare-die and packaged die to quantify the shifts of the first resonance frequency of the CMUT.

**Figure 6:**
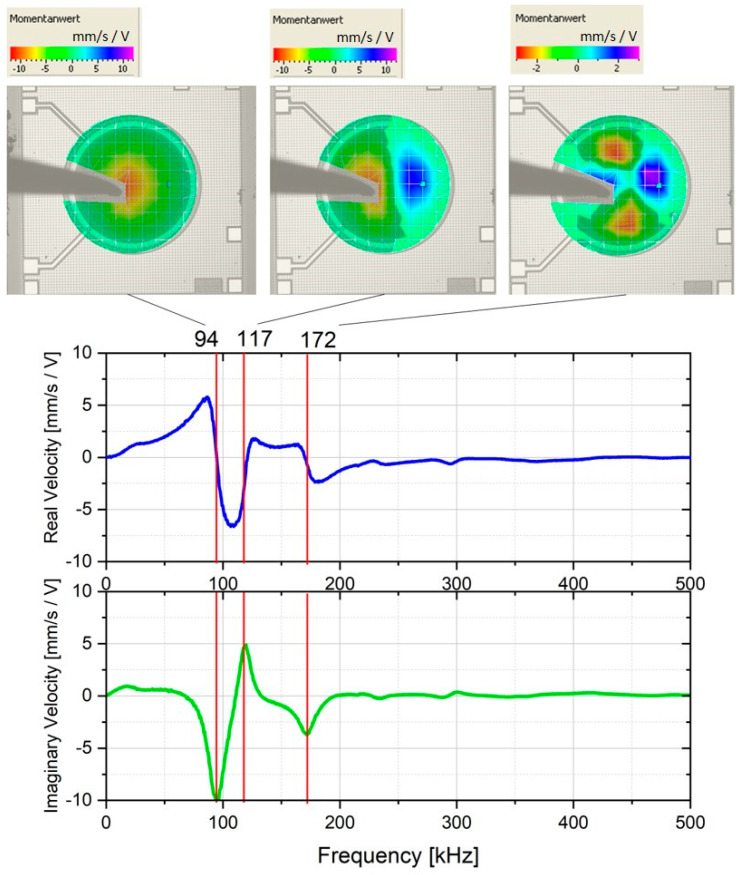
Averaged laser-Doppler vibrometry (LDV) measurement results of the bare CMUT dies with the images of the modes shown above at the characteristic frequency.

**Figure 7:**
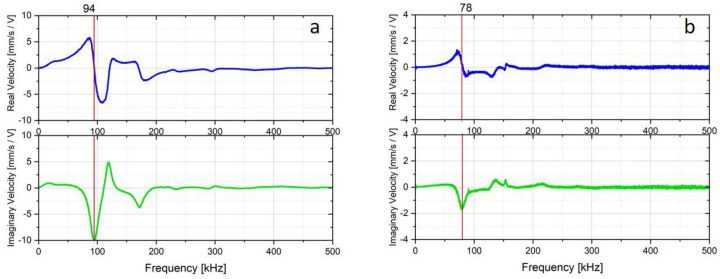
A comparison between the averaged LDV-Measurements of CMUT die before (**a**) and after (**b**) packaging indicating a shift in the first resonance frequency of ca. 16 kHz.

Figure 6 shows the result of the average LDV-measurement for the bare dies. The response curves of the membranes were monitored and averaged over several measuring points, which were positioned as an overlaid grid. The speed frequency response with its real (blue) and imaginary (red) part is shown in the graphs. When the imaginary part had an extremum and the real component had a turning point, a characteristic mode was formed. The averaged LDV measurement result of the packaged dies is also shown and compared with the bare-dies in Figure 7. The first resonance frequency of the membrane was measured after packaging with a value of 78 kHz. As inferred from Figure 6 and Figure 7, there was a shift in the first resonance frequency caused by the packaging of ca. 16 kHz. This shift was attributed to the compressive stresses due to the chemical and thermal shrinkage (CTE mismatch) of the encapsulation material after cooling down from molding temperature to room temperature. It was postulated that the inkjet printing of RDLs could not have a remarkable influence on the induced stress, since the curing temperature of the inks is identical to the post-mold curing temperature of 150 °C. Besides, neither pressure, aggressive chemical treatment nor a physical contact to the CMUT membranes was imposed during inkjet printing. It is noteworthy to mention that in the course of FOWLP, after compression molding, two temperature-assisted processes were exerted, i.e., post-mold curing at 150 °C and removal of the release tape at 180 °C. 

### 3.3. Reliability Analysis

**Figure 8:**
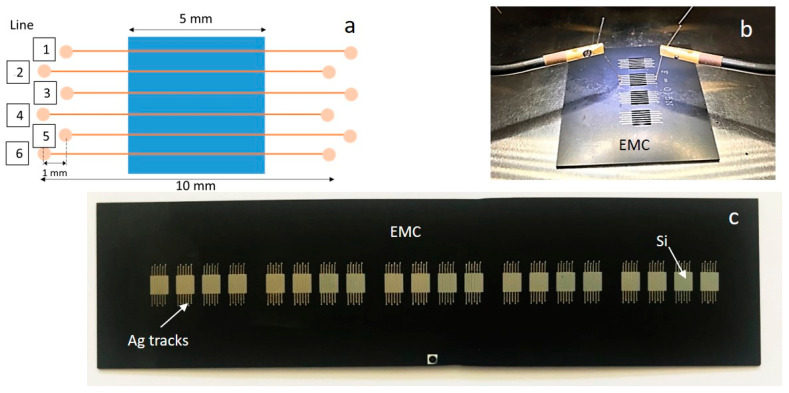
Schematic (**a**) and experimental setup (**b**) for temperature cycling test. An example of the test sample arrays for temperature cycling test is shown in (**c**).

Accelerated tests are often used to get a deeper understanding of the reliability of components and the collaboration of those within a system. Since the proposed technology (developed within the Silense project [8]) was aimed for mobile and automotive applications, temperature cycling according to the automotive electronics council (AEC-Q100) Grade 1 standard was selected as the main verification methodology. Consequently, the temperature profile was selected according to the standard (−40 to 125 °C) with a time course of a cycle being 130 min with a 30 min holding phase at each peak temperature [24]. Here, a new set of samples was designed and fabricated. The simplified design and final configuration of the test samples for reliability analysis are shown in Figure 8. As seen, the test samples comprised embedded Si chip arrays with 6 printed Ag tracks per chip. Electrical characterization via a two-wire-method was conducted beforehand and at defined intervals during testing at room temperature. 

**Figure 9:**
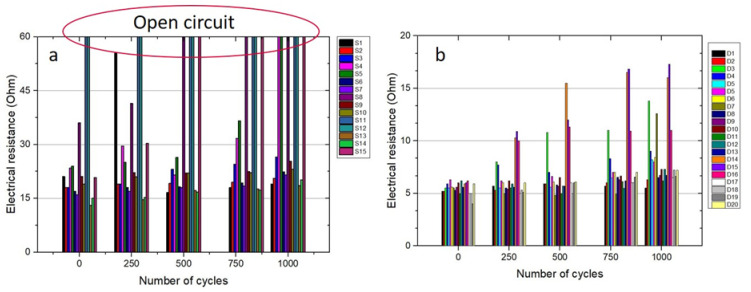
Temperature cycling test results of single-layered (**a**) and double-layered (**b**) Ag tracks.

In Figure 9, the measured resistances of the single and double layer printed conductors are compared. It can be seen that the average electrical resistances of the double layer ones were lower than that of the single layer. Moreover, in the course of thermal cycling, the double-layered lines exhibited more consistency compared to the single-layered lines. As highlighted in the graphs, several open circuits emerged during thermal cycles of single layers. 

It was postulated that the thin layers are prone to higher reliability issues since the thin single layer cannot accommodate the thermomechanical stresses due to the CTE mismatch between EMC (~7 ppm/K) and sintered ink (~19 ppm/K) during the thermal cycle tests. There was also a risk that a single-layer Ag could not fully cover the 3–6 µm step-height between the chip and the EMC. The surface roughness (arithmetic mean of the measured absolute height, Sa) of the EMC was measured to be in the range of 450–603 nm, given that a double-layered Ag layer with a thickness of 2 µm seemed to be a more reliable approach to provide a homogeneous layer all over the surface compared to a single layer with a thickness of less than 1 µm. Conclusively, the double-layer printing of Ag lines was proposed to be the best compromise between the reliability, process speed and the final line-width of the RDLs.

**Figure 10:**
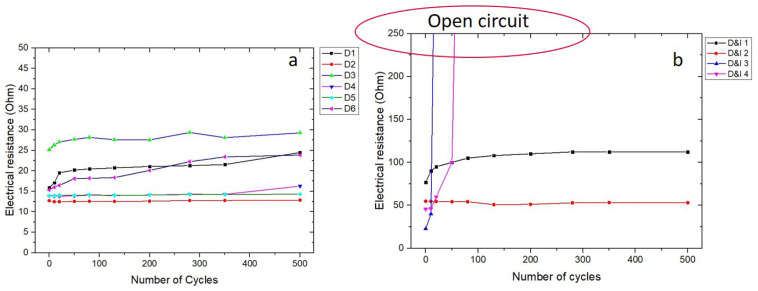
Temperature cycling test results of double-layer Ag lines without (**a**) and with (**b**) insulating ramp.

In the second set of experiments, the effect of the dielectric ramp on the reliability of the printed interface between die and EMC was investigated. The results of the thermal cycle tests of the samples with and without the insulating ramp are compared in Figure 10. In contrast to our expectation, the insulating ramp did not improve the performance of the printed lines, but rather increased the total electrical resistance of the tracks. There were also more cases of open circuits found. A possible explanation for this observation was the higher CTE of the dielectric polymer inks, which induced additional thermomechanical stresses during thermal cycling. In fact, as to keep the viscosity of the ink low, the inkjettable dielectric materials usually do not contain any fillers and thus possess high CTEs [16,17,25]. In another study, SU8 dielectric ink was inkjet-printed as an insulating ramp to generate 3D interconnects for a millimeter-wave system-on-package [26]. The used SU8 had a CTE of about 52 ppm/K [27]. The inks employed in the current study were also supposed to have similar CTEs, although these values were not provided by the material suppliers.

**Figure 11:**
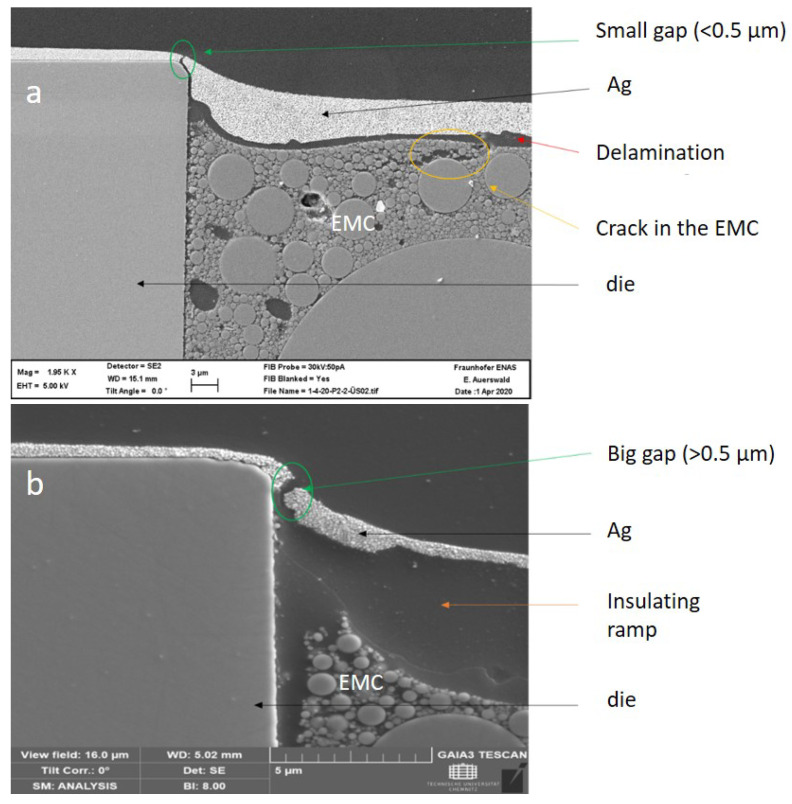
Cross-sectional scanning electron microscopy (SEM) images of the broken Ag tracks at the interface of EMC/die after thermal cycling manifesting the hotspots of the interconnects; (**a**) the sample with double-layered Ag line (**b**) the sample with double-layered Ag line and insulating ramp.

The cross-sectional images of two failed samples with and without insulating ramp after 500 cycles are presented in Figure 11. It is evident that the fracture took place at the interface between the die and EMC. This interface was subjected to the fusion of thermomechanical stresses due to CTE mismatches between different materials, i.e., Ag/EMC/Si or Ag/EMC/dielectric ramp/Si. It can be seen that the gap was broader in the case of the insulating ramp approach, which is consistent with the measured electrical resistance results.

Conclusively, it can be deduced that the double-layer Ag layer yielded more reliable interconnects compared to single-layered interconnects or interconnects with the insulating ramp. It was also found that the step-height between the chip and EMC led to reliability issues and should be minimized. The magnitude of this height difference is assumed to be dependent on the CTE mismatch between the EMC and the dies, thus also on the thermal budget throughout the manufacturing process. Chemical shrinkage of the EMC impacts this further, as well as the choice of temporary adhesive (thermal release tape) and possibly the placement force. The resulting steps, even though only a few µm high, was identified as a potential bottleneck for the printed lines, especially considering reliability performance. Additionally, the observed delamination in Figure 11a implied a degraded adhesion between the Ag and EMC upon temperature cycling. By comparing Figure 11a to Figure 3d, one can deduce that the delamination emerged during the thermal cycling test due to the CTE mismatch. 

### 3.4. Barriers to Overcome

This study sheds light on challenges and opportunities in FOWLP of CMUT arrays by using inkjet-printed RDLs. It can be inferred that inkjet printing is a cost-effective, powerful and rapid way to form RDLs, especially in comparison to conventional lithography- and electrochemical-based formation of the RDLs. It is well-suited for MEMS packaging, as the additive manufacturing of the RDL eliminates all sorts of challenges with delicate sensing surfaces (i.e., fragile membranes). MEMS also have typically few I/Os; thus, a low-density FOWLP with larger line-width within the resolution of inkjet printing (30–100 µm) can be feasible.

The challenges can be divided into inkjet-related and FOWLP-stemmed issues. There are still some crucial issues that can hinder the full implementation of inkjet printing for FOWLP packaging, such as the poor conductivity of the metallic inks, limitations in resolution for high-density FOWLP, and signal integrity for high frequencies as well as the reliability issues. One of the major limitations of inkjet-printing lies in the strict rheological requirements of the inks, i.e., small range of viscosity and surface tension. Additionally, the coffee-ring effects due to the uneven drying of the inks are still an issue with inkjet-printed structures [28]. Despite the current limitations, with the advancement of additive-manufacturing processes and improved materials, digital printing can certainly overcome the existing challenges. For instance, the current line-width limitations and rheological requirements can be overcome using other printing approaches such as electrohydrodynamic inkjet printing [29,30]. 

Concerning FOWLP, the encapsulation of delicate CMUT components for FOWLP was found to be challenging, since stress management and protective concepts for sensing areas were determining overall system performance. In addition, the Z-offset issue, which was found to be the reason for the reduced long-term stability, should be further investigated and mitigated upon the consequent process and material optimizations. Future work can be also devoted to improving the adhesion of the Ag ink to EMC by employing different surface pretreatments.

## 4. Conclusions

In this study, an innovative approach for FOWLP of CMUT sensors by using inkjet-printed redistribution layers was pursued. Two realizations of the proposed FOWLP were shown and the performance of the packaged sensors with sensitive membranes was compared to bare dies performance. The reliability of the printed RDLs was assessed by using a thermal cycling test. The effects of multilayering and incorporating an insulating ramp between the die and mold were investigated. The cross-sectional analysis of the failed samples manifested the bottlenecks of the printed lines. Consequently, the challenges and opportunities of printed RDLs were addressed. The proposed approach for FOWLP of MEMS by using inkjet printing could eventually lead to a new platform for cost-effective heterogeneous integration.
